# Pandemic lockdowns: who feels coerced and why? - a study on perceived coercion, perceived pressures and procedural justice during the UK COVID-19 lockdowns

**DOI:** 10.1186/s12889-024-17985-1

**Published:** 2024-03-13

**Authors:** V. Ranieri, C. Gordon, S. K. Kamboj, S. J. Edwards

**Affiliations:** 1 Research Department of Clinical, Educational and Health Psychology, University College London, London, UK; 2 Department of Science, Technology, Engineering and Public Policy (STEaPP), University College London, London, UK

**Keywords:** Perceived coercion, Perceived pressures, Procedural justice, Fairness, Choice, COVID-19, Lockdown

## Abstract

**Background:**

This study examined perceptions of coercion, pressures and procedural injustice and how such perceptions influenced psychological well-being in those who experienced a UK COVID-19 lockdown, with a view to preparing for the possibility of future lockdowns.

**Methods:**

40 individuals categorised as perceiving the lockdown(s) as either highly or lowly coercive took part in one of six asynchronous virtual focus groups (AVFGs).

**Results:**

Using thematic analysis, the following key themes were identified in participants’ discussions: (1) Choice, control and freedom; (2) threats; (3) fairness; (4) circumstantial factors; and (5) psychological factors.

**Conclusions:**

As the first qualitative study to investigate the psychological construct of perceived coercion in relation to COVID-19 lockdowns, its findings suggest that the extent to which individuals perceived pandemic-related lockdowns as coercive may have been linked to their acceptance of restrictions. Preparing for future pandemics should include consideration of perceptions of coercion and efforts to combat this, particularly in relation to differences in equity, in addition to clarity of public health messaging and public engagement.

**Supplementary Information:**

The online version contains supplementary material available at 10.1186/s12889-024-17985-1.

## Background

Perceived coercion, perceived pressures and procedural justice are psychological constructs used to indicate the degree to which an individual perceives they are able to exercise choice, autonomy and control over circumstances they experience as fair and occurring free from force or threat [1, 2]. Historically used to describe a mental health service user’s experiences of admission to hospital, high levels of perceived coercion have previously been linked to poorer treatment outcomes, greater dissatisfaction with mental health services, diminished adherence to treatment and increased disengagement from mental health services and treatment [2–4].

The above constructs may apply to other circumstances involving restrictions on people’s movement and certainly to physical confinement that have some similarities to detention in hospital. An example of such circumstances is quarantine or ‘lockdown’ when containment through contact tracing is no longer feasible during severe public health emergencies. Implemented in response to increased rates of transmission of COVID-19 and pressures on national hospital capacity, lockdowns in the UK mandated citizens to remain within their homes except to collect essential goods, exercise, receive clinical care, or travel to an essential workplace [5]. The UK’s first lockdown took place between 26th March and 23rd June 2020 followed by sporadic local lockdowns and a three ‘tier’ system based on incidence rates. A subsequent national lockdown took effect from 5th November for four weeks merging into a three then four ‘tiered’ system before another national lockdown on 6th January 2021 and phased return between March and July 2021. A recent scoping review on perceived coercion within the context of the COVID-19 pandemic, gave an early indication that many individuals met lockdowns with an initial acceptance that decreased over time as a sense of intrusiveness by authorities emerged worldwide [6, 7]. It also highlighted a link between low perceived control and greater depressive and anxious symptomatology [8], As found in the scoping review, it remains unclear as to who perceives lockdowns as more coercive and procedurally unjust and whether there are specific individual experiences and characteristics that increase the likelihood of these perceptions and of potentially dissatisfaction and disengagement with restrictions [7].

The present study aimed to better understand such perceptions and test whether the psychological constructs and measures related to perceived coercion may fit scenarios that involve the deprivation of liberty of the general population in the context of widespread international emergency. Using a qualitative methodology, it examined the views of those who experienced a COVID-19 lockdown in the UK in relation to perceived coercion, perceived pressures and procedural justice by means of thematic analysis. Using multiple asynchronous virtual focus groups (AVFGs), it aimed to describe the factors that influenced participants’ perceptions regarding the lockdowns and how these may have impacted on or been impacted by participants’ psychological well-being, with a view to preparing for the possibility of future lockdowns.

## Methods

### Recruitment

Participants were recruited from an online survey conducted by the authors on the topic of perceived coercion and psychological wellbeing arising from lockdown [9]. Participants were recruited to the online survey between 22nd of July 2020 and 3rd October 2020 (covering first national and several local city lockdowns) via advertisements posted on Facebook, Reddit, Twitter, and Instagram. Although we employed convenience sampling, recruited participants were from various geographical and cultural communities, and COVID-19 support groups across the UK. A total of 2,003 individuals took part in the online survey (the parent survey). Of these, 1,026 entered their email address on the final page of the survey indicating that they agreed to be contacted to take part in further research. The AVFGs took place in April 2021. This followed the re-opening of schools in March 2021 and coincided with the re-opening of non-essential retail and public offices mid-April, with individuals being allowed to meet in small groups (of six) the following month (May 2021).

### Participants

40 participants individuals took part in the study. They consisted of adults aged 22–76 years who experienced the UK governmental lockdowns. Most were White females based in England. The primary inclusion criteria for the study were age ≥ 18 years, resident in the UK at the time of the lockdowns and felt comfortable writing about their viewpoints in English. Participants were purposively sampled according to age and gender (where possible) and divided into two categories according to whether their responses, as per the amended MacArthur Admission Experience survey (AES) included within the parent survey, indicated that they perceived the lockdown as coercive to a high (scores of 4–5) or low degree (scores of 0–1) [1]. There were 6 AVFGs consisting of 6–9 participants in each: four groups included individuals who did not perceive the lockdowns as coercive and two groups involved individuals who viewed the lockdowns as very coercive.

### Setting

AVFGs were hosted on UCL Extend, a Virtual Learning Environment supported by UCL typically used to host short courses and professional education. This platform was chosen as a secure and confidential space for research participants to widen access to participants who could log on at any time of the day or night in order to write and interact with each other.

### Procedure

Participants who agreed to be contacted following the parent online survey were sent an email inviting them to take part in this qualitative study. Those interested were invited to register under an alias in order to preserve their anonymity. Informed consent was registered within the platform. Upon entering the AVFG, the first focus group questions appeared as a discussion topic. Participants were asked to post their responses to a different set of questions each week for three weeks, relating to their views and experiences of the lockdowns. The questions (please Table 1) were formulated as a result of some of the more prevalent topics elicited within the parent survey and followed the transactional theory of stress and coping (linking appraisal or ‘perceptions’ to psychological wellbeing) [10]. Participants could also comment on other focus group members’ posts. Discussion boards were checked twice daily in order to monitor participants’ wellbeing during the study and to re-direct them to the appropriate services in case of significant distress.

### Analysis

A phenomenological approach was applied to the study to better understand participants’ subjective viewpoints of the lockdowns and how these were shaped by their experiences and personal circumstances. Focus group data were downloaded directly as text from UCL Extend and coded using NVivo as a data management system [11]. Braun & Clarke’s (2006) thematic analysis was used to analyse texts by grouping commonalities and experiences between groups on individuals [12]. Themes were explored independently by two researchers (VR and CG), and any disagreements discussed and resolved.

**Table 1:** Focus group questions divided according to week

Week	Question(s)
Week One	*Your views of the lockdown(s)* Last week marked one year since the first lockdown came into place. Looking back, how did you feel when the first lockdown was announced? Have those feelings changed since then? If yes, in what way? What, for you, were the advantages and disadvantages to the first and subsequent lockdowns?
Week Two	*Perceived Coercion: The extent to which we feel we have choice, freedom, and control over our lives* From our survey, some individuals reported feeling coerced as they experienced a loss of choice, control and freedom over their lives due to the lockdown(s). Reflecting on your own personal circumstances (i.e. living, work, family or other circumstances), what factors played a role on how you viewed the lockdown? How did those personal circumstances impact on whether you perceived the lockdown as coercive?
Week Three	*Your Mental Health* Welcome to our final week! Last week we pondered about the extent to which you felt free and in control of your life during lockdown and how this feeling was impacted by your own personal circumstances. This week, we’ll delve into your emotional wellbeing instead. How has the lockdown impacted on your mental health? What have you done so far to try to stay well? How would the announcement of a future lockdown impact on your mental health? What support (psychological, physical or other) do you feel you need now for your emotional wellbeing? What support would you benefit from in the future should this be announced?

## Results

The following key themes were central to participants’ discussions: (1) Choice, control and freedom; (2) threats; (3) fairness; (4) circumstantial factors; and (5) psychological factors. Results from both those in high and low perceived coercion groups have been combined and identified within each theme.

### Perceived coercion: choice, control and freedom

All participants spoke of initially accepting restrictions “*for the greater good*” in order to avoid spreading the virus and protect those most vulnerable within society. Most also spoke of growing tired of restrictions. Many of those who perceived the lockdowns as more coercive (i.e. scored high on perceived coercion) also spoke of feeling controlled by the state who mandated unclear and ever-changing restrictions with no opportunity to express their apprehension or contribute to decision-making. Some spoke of feeling infantilised by and resentful of restrictions that, in their eyes, did not address socioeconomic difficulties that impacted on rates of transmission, such as overcrowding, poor working conditions and poverty. These also spoke of feeling oppressed, particularly when living alone and lacking social contact, and of having little choice or freedom over their lives more broadly. Some of these spoke of being prevented from determining their “*own level of acceptable risk*” and feeling that the virus presented little risk of death to them.

*“I really hate the concept of not being “allowed” to do things, as though we are children who cannot be trusted to make our own decisions.”* Participant PS

*“Yes, I did feel it was coercive. I would have rather we had been asked to comply with covid regs rather that there being laws in place to force people.” Participant BB.*

Contrastingly, the vast majority of those who did not perceive the lockdowns as coercive spoke of *choosing* to restrict their behaviours but also accepting a need for less choice in order to safeguard their community. These spoke of agreeing with the lockdown as a necessary step to prevent deaths and believed that lockdown was in their best interest and that protecting the NHS was a ‘duty’. Some stated that they felt reassurance and relief when the lockdown was announced though others felt it was implemented later than preferred. Fear of contracting COVID-19 motivated a large proportion of this group to adhere to restrictions, with some expressing that they lived more cautiously than outlined by the regulations at the time and isolated prior to lockdown starting.

*“My primary concern has been to be part of the wider societal effort to help protect as many people as possible from contracting this disease, whether they be in my own household or strangers to me. It is this feeling that makes it feel less like coercion and more of a choice.”* Participant AA.

*“We still had the ability to make choices they were just lessened. I felt that the Government were only acting in our best interests. It wasn’t about being in control. It was about being safe and protecting the most vulnerable and in order to do that if I had to put my life on hold for a year or so then there was no question that I wouldn’t do it. I was terrified of catching the disease and can imagine for the vulnerable it was much more of a scary prospect.”* Participant RW.

Many participants in both high and low PC groups found some of the restrictions fatuous. Those who did not perceive the lockdowns as coercive were comfortable “bending” the rules to make lockdown more “bearable” where these were judged not to increase risk of transmission. Examples of adapting the rules were exercising multiple times a day, forming support bubbles with isolated individuals, and meeting friends and family in the garden or outdoors.

*“So I suppose another reason I didn’t feel coerced was that I felt at liberty to ignore the rules if I judged I wasn’t doing any harm by doing so… I have to own up that I did break the rules. When we were only meant to go out for exercise once a day, I often went out twice– for a run by myself in the early morning then a walk with my partner later in the day. I knew this was not allowed but cleared it with my conscience as I seldom met anyone on these outings, and if I did I kept well apart from them. I didn’t feel I was causing any more danger than if I’d only gone out once.”* Participant MM.

In comparison to those who experienced lockdown as more coercive, a minority of those with lower perceived coercion scores tended to report feeling coerced by a slow response from governments to the spread of COVID-19 and a UK-specific lack of provision of public health education. These also expressed dismay at English governmental policies they viewed as posing an increased risk of transmission. A minority of these expressed that restrictions and the policing of these were too permissive. Discussions regarding mandatory vaccination also highlighted a perception of control by the state in participants in both groups, with a small proportion viewing it as a way of regaining control over transmission.

*“I feel many people have been coerced into irrational behaviour through perverse policies such as “eat out to help out”, which are clearly economically motivated… it seemed obvious to me that the lack of action from the vast majority of countries in the world to prevent the spread was turning the then epidemic into a pandemic. It would not have been my choice, and I do feel that I have been coerced into living for years through an unnecessary pandemic… Rules are always blunt instruments and if we are going to keep infection rates down we need to move from rules to educating our nation, so that people can make good individual choices. I view the government’s failure to do this as a form of coercion. There has not been a significant public health education effort from the government to teach people about the relative risks of surface vs airborne transmission, of the basic science behind improving ventilation, of methods for reducing virus concentrations”* Participant DD.

### Perceived pressures: threats

Participants from both groups spoke of penalties being issued to individuals for non-adherence to restrictions. Many of those who felt more coerced during lockdown tended to view these as threats and voiced concerns regarding the police’s power to impose fines for ordinary daily activities, such as walking, under the rubric of ‘non-compliance of restrictions’. These also expressed concern that public messaging via governmental campaigns led people to see others’ actions, rather than the virus itself, as a source of threat that encouraged public shaming. Those who did not perceive the lockdown as coercive tended to, mostly, either agree with the use of penalties or view them as a nuisance or legal penalty to be avoided rather than a threat.

*“This was all underpinned and maintained through threats. We had the threat of being fined by police for any non-compliance with the regulations, and later the threat of being refused entry or service by premises if face covering requirements weren’t adhered to. Judgment was also used, with (as just one example) the Met Police Commissioner asking the public to shame others for not wearing face coverings. The virus itself also became used as a threat. Announcements on the TV and newspapers warned us that, at different times, things like meeting up with a friend or sitting on a park bench would cause people to die. The “look in their eyes” advertising campaign was the most egregious example, implying that people were in hospital not so much because they’d caught a highly transmissible respiratory infection, but because of the harmful actions of others.”* Participant TP.

*The police were picking on anyone they felt would be an easy target… and certain types of people were taking it upon themselves to enforce their own version of the rules on their neighbours by intimidation and threats. The atmosphere began to change from the original ‘Blitz Mentality’ of we are all in this together to something more akin to 1930’s Germany where the message was ‘Comply and Conform or Else We are Coming For You’.* Participant DZ.

*“I haven’t seen the lockdown as coercive. Or no more coercive than a speed limit or any other legal boundary which has been put in place for safety reasons. Disobeying wasn’t physically impossible for me, nobody locked me in my house, and I honestly think not wanting the stress of being challenged by the police was a bigger incentive to compliance than the actual fines… The idea of a family member or anyone else dying as a result of my actions is much more compelling to me than a fine.”* Participant PE.

A minority of individuals in both groups also conveyed significant concern about the possibility that the governmental policies that placed temporary restrictions on citizens’ freedom of movement could pose a threat to their and future generations’ human rights.

*“Although I haven’t felt myself to be coerced so far… there are moves towards changing the frameworks that protect our democratic rights and freedoms. I don’t doubt that emergency powers are necessary at an exceptional time– what does worry me, though, is that when the pandemic comes to an end we might unwittingly find ourselves in a more coercive, less humane society than we ever anticipated.”* Participant II.

Additionally, some individuals spoke of experiencing threat in relation to their job security whereby employers were pressuring these to be in the workplace despite not fully recovering from COVID-19 and feared being unable to find another job.

### Procedural justice: fairness

Such perceptions of coercion were amplified in participants who felt that lockdown was applied unfairly across the UK and who lived in areas where restrictions were longer in duration. Those who lived in hotspots where lockdowns lasted longer in England, spoke of being unsure of the strength of the evidence drawn upon to inform the severity of the restrictions and felt that reporting of transmission rates in those areas were being manipulated or used as a ‘cautionary tale’.

*“When we had the tiers system, it felt as though the goalposts were always changing– the numbers of cases per 100,000 people that were sufficient to put large regions of the north of England into tier 3 were seemingly not enough to bring London and other areas of the south east out of tier 2. If a particular number had been decided upon as the threshold for tier entry and then consistently applied regardless of geography, that would have been a much fairer approach.”* Participant FM.

*“I have lost a lot of freedom over the last year and at times felt like a prisoner at home - and the Leicester lockdown really felt like we had been forced into a position that was unfair and we lost control of our lives. However as time went on and due to living in Leicester I began to feel like it was political, it was a way to control a city but really was largely ineffective - the fact that the Government wouldn’t play ball locally with releasing data on what was needed to get us out of lockdown, made me feel that Leicester was used as an example of what happens if your numbers go up.”* Participant RR.

A sense of community spirit was shared by many in both groups. Multiple individuals in both groups made comparisons to World War II and the need for communities to support each other. These also shared a sense of unfairness in relation to regulations that violated their values or appeared to be ill-evidenced. Examples of these pertained to restrictions on the number of attendees or proximity between attendees at funerals, and visitation rights in care homes.

*“I do feel that we’ve lost some of our humanity during this lockdown. The footage of a son at a funeral, who’d moved his chair to be closer to comfort his mum and an official telling him off and to move apart was especially inhumane… Where was the evidence for there to be only 6 at funerals. It was cruel and unnecessary. I would have thought that numbers should depend on the size of the room so that social distancing could be maintained.”* Participant BE.

Many individuals within both high- and low-perceived coercion groups expressed distrust in government. Some expressed concern over nepotism and mishandling of procurement contracts relating to personal protective equipment and technologies for tracking and preventing transmission (i.e. test and trace), the ethicality of campaigns that they perceived as helpful towards regrowing the economy though likely to raise rates of transmission (e.g. the government subsidised ‘eat out to help out’ campaign). Most participants across the two groups also spoke of perceiving the government as not abiding by the rules it set for its citizens and wrote of perceiving the government’s actions as increasingly authoritarian. Some also spoke of perceiving decisions made by the government as ‘out of touch’ with their everyday life experiences due to a difference in socioeconomic privilege between cabinet members and the general population. For some, such perceptions of the government contributed to their view of lockdown as coercive whilst others viewed the lockdown as a needed step to mitigate against the threat of the virus, regardless of political action.

*“I have little faith in certain members of the government, but I saw the move to close things down as a sign of the huge science-backed threat the virus posed to us all and the NHS, rather than coercive.”* Participant JH.

### Circumstantial factors

Some participants’ perceptions of the lockdown appeared to be shaped by their own socioeconomic circumstances and opportunities. Many of those who did not perceive the lockdowns as coercive spoke of being able to live at home with relative ‘ease’ due to being able to work from home, being retired, not experiencing economic hardship or being comfortable at home alone or supported by immediate family. Some of these wrote of ‘enjoying’ lockdown and being able to relax outside of the constraints of prior ‘normal’ life.

*“I felt very conscious of my good luck which made it easy for me to comply with the rules. I have a garden, live within walking distance of countryside, and was with my partner who is also my best friend and my son who is good company.”* Participant MM.

Others longed for their prior lives. Women in both high and low perceived coercion groups conveyed feeling a lack of control over their lives and an increased level of distress due to having little support in managing childcare or other caregiving responsibilities whilst working in lockdown.

### Psychological factors

The majority of participants across the groups spoke of feeling anxious and worried regarding their own health, the possibility of loved ones catching COVID-19, and uncertainty regarding the future. Many also spoke of experiencing low mood due to increased loneliness and isolation from reduced contact with extended family and friends or from having limited freedom to carry out activities that they previously enjoyed (e.g. travel and exercise). Participants noted that the second lockdown which took place in Winter felt more difficult as opportunities to be outside were reduced and as lockdown no longer presented itself as a novelty. Some expressed hopelessness in relation to ongoing lockdowns and potential future restrictions. Contrastingly, those in the high perceived coercion group wrote of feeling powerless, with some feeling that the lockdown exceeded their coping reserves and that their views didn’t coincide with those of others’ they knew. Those in the low perceived coercion group wrote of “*feeling part of something bigger*” and focusing on what they could control in their everyday lives. When asked about what support would aid their psychological wellbeing, both groups expressed a need for the relaxing of restrictions and, for some, the availability of counselling.

*“Not only did I feel stressed and depressed, I actually think I felt disassociated; I couldn’t believe that governments around the world were sticking with lockdowns in spite of all the evidence and data that the virus was nowhere near as bad as first claimed, and it got to the point where I actually wondered on more than one occasion if I’d totally lost my mind, if I was imagining the things I’d read because it was so at odds with the government position.”* Participant TP.

## Discussion

To the authors’ knowledge, this is the first qualitative study explicitly examining the superordinate psychological construct of perceived coercion (incorporating perceived pressures and procedural justice) in relation to COVID-19 lockdowns during a global health emergency. Though participants spoke of initially viewing the lockdown as a way of reducing the spread of the virus, those who found the lockdowns more coercive conveyed feeling ‘controlled by the state’ and feeling infantilised by being unable to determine and act according to their personal perceived level of acceptable risk to the virus. Conversely, those in the low perceived coercion group wrote of *choosing* to follow restrictions and accepting that they would temporarily experience some limitations over their day-to-day lives in order to protect their community. Of note, were participants’ differences in perceptions with regard to threat with those in the low perceived coercion group viewing illness as a primary threat and the majority of their counterparts in the high perceived coercion group viewing the implementation and policing of the lockdowns, rather than the illness, as the primary threat. Subthemes of distrust in government, authoritarianism, a sense of unfairness regarding the unequal geographical implementation of restrictions and the disproportionate impact these had on individuals with less socioeconomic privilege were highlighted within both groups. Whilst powerlessness and difficulty coping with ongoing restrictions featured in the accounts of those who viewed the lockdowns as more coerced, focusing on what participants could control in their day-to-day lives was described by those who felt less coerced.

Though the topic of perceived coercion is unexplored within the literature on public responses to pandemics, this study supported prior COVID-19 related findings that greater perceived control over one’s circumstances during lockdowns was linked to willingness to adhere with restrictions and that those who viewed themselves as having less decision-making power over their circumstances were less likely to adhere to such [13–17]. Our findings also support prior research that highlighted that willingness to lockdown may have been linked to individuals’ perceptions of political leadership and fairness in leaders’ actions [18] This study also supports prior research that found that individuals ‘bent’ lockdown rules to make these more digestible, particularly when individuals felt more socially isolated [19]. It also upheld the findings of a prior study that found that individuals who perceived others as insufficiently complying with restrictions were in favour of greater pandemic-related social control [20]. Participants spoke of experiencing anxiety and low mood linked to lockdown and a sense of ‘entrapment’ in this and previous studies [8, 21]. Unlike a previous scoping review by the authors, perceived pressure in the form of social norms and perceived control in the form of conspiracy theories did not feature within this study [7].

The study included both strengths and limitations. A strength of the study pertained to the use of AVFGs that *potentially* widened access and geographic representation of participants, and allowed flexible participation at any time of day/night. The use of AVFGs also allowed participants to take part in the study anonymously. Limitations of the approach included the requirement for English literacy and access to and competence in the use of technology. As participants elected to participate in the study, it is possible that some of those who participated may have had more time availability and stronger opinions which may have introduced self-selection bias. A further limitation was that despite using an approach that was *potentially* demographically inclusive, our sample were mostly White and female. Thus, the extent to which the identified themes are relevant to a diverse population is therefore unclear.

## Conclusions

Our findings indicated that the extent to which individuals perceived pandemic-related lockdowns as coercive may have been associated with their acceptance of restrictions. preparing for future pandemics should include consideration of perceptions of coercion and efforts to combat this, especially where such coercion is experienced or perceived as being illogical or applied inequitably. It is understandable that some UK residents in lockdown who were asked to temporarily behave collectivistically to protect others, whilst based in a culture that typically promoted lifelong individualistic and capitalist values outside of a pandemic, experienced psychological distress. Therefore, it is possible that our findings may not apply in more collectivist cultures.

The way the restrictions and changing public health measures are explained and communicated, though, requires closer study to reduce risk of appearing authoritarian or illogical and to motivate continued commitments to protect others when faced with outbreaks of severe infectious diseases. These would need to emphasise what choices remain available within those restrictions, clarify apparent inconsistencies and counterintuitive measures, sometimes a result of oversimplify messaging, and avoid apparent toxic or manipulative strategies such as playing on fear or encouraging neighbourhood shaming.

Future public health campaigns may, therefore, alleviate some of these concerns by focusing on public health education designed to engage with the public as understanding of the virus grows and priorities shift, rather than simply impose heavy blanket restrictions with growing threat of sanctions, sometimes applied in seemingly arbitrary fashion, for the sake of maintaining social law and order and policing by consent. As the UK Public Inquiry continues to hear evidence from politicians, science advisors and grieving families, it is clear than we still have much to learn to be better prepared next time the country faces a public health crisis of this sort.

### Electronic supplementary material

Below is the link to the electronic supplementary material.

Supplementary Material 1

## Data Availability

The datasets generated and/or analysed during the current study are not publicly available due to preserve participants’ anonymity but redacted versions are available from the corresponding author on reasonable request.

## References

[1] Gardner W, Hoge SK, Bennett N, Roth LH, Lidz CW, Monahan J (1993). Two scales for measuring patients’ perceptions for coercion during mental hospital admission. Behav Sci Law.

[2] Lidz CW, Mulvey EP, Hoge SK, Kirsch BL, Monahan J, Eisenberg M (1998). Factual sources of psychiatric patients’ perceptions of coercion in the hospital admission process. Am J Psychiatry.

[3] Katsakou C, Bowers L, Amos T, Morriss R, Rose D, Wykes T (2010). Coercion and treatment satisfaction among involuntary patients. Psychiatric Serv.

[4] Kaltiala-Heino R, Laippala P, Salokangas RK (1997). Impact of coercion on treatment outcome. Int J Law Psychiatry.

[5] Mahase E (2021). Covid-19: NHS test and Trace failed despite eye watering budget, MPs conclude. BMJ.

[6] Bernacer J, García-Manglano J, Camina E, Güell F (2021). Polarization of beliefs as a consequence of the COVID-19 pandemic: the case of Spain. PLoS ONE.

[7] Ranieri V, Kamboj SK, Edwards SJL (2023). Perceived coercion, perceived pressures and procedural justice arising from global lockdowns during the COVID-19 pandemic: a scoping review. PLOS Glob Public Health.

[8] van Mulukom V, Muzzulini B, Rutjens BT, van Lissa CJ, Farias M (2021). The psychological impact of threat and lockdowns during the COVID-19 pandemic: exacerbating factors and mitigating actions. Translational Behav Med.

[9] Ranieri V, Sem Stoltenberg A, Pizzo E, Montaldo C, Bizzi E, Edwards S (2021). COVID-19 welbeing study: a protocol examining perceived coercion and psychological well-being during the COVID-19 pandemic by means of an online survey, asynchronous virtual focus groups and individual interviews. BMJ Open.

[10] Lazarus R, Folkman S, Stress (1984). Appraisal and Coping.

[11] QSR International. NVivo. Melbourne, Australia: QSR International; 2023.

[12] Braun V, Clarke V (2006). Using thematic analysis in psychology. Qualitative Res Psychol.

[13] Ceylan M, Hayran C. Message Framing Effects on Individuals’ Social Distancing and Helping Behavior During the COVID-19 Pandemic. Front Psychol. 2021;12. 10.3389/fpsyg.2021.579164 PMC8019916 33828501

[14] Frounfelker RL, Santavicca T, Li ZY, Miconi D, Venkatesh V, Rousseau C (2021). COVID-19 experiences and Social Distancing: insights from the theory of Planned Behavior. Am J Health Promotion.

[15] Hills S, Eraso Y. Factors associated with non-adherence to social distancing rules during the COVID-19 pandemic: a logistic regression analysis. BMC Public Health. 2021;21(1). 10.1186/s12889-021-10379-7 PMC7881344 33581734

[16] Lo Presti S, Mattavelli G, Canessa N, Gianelli C (2022). Risk perception and behaviour during the COVID-19 pandemic: Predicting variables of compliance with lockdown measures. PLoS ONE.

[17] Sobkow A, Zaleskiewicz T, Petrova D, Garcia-Retamero R, Traczyk J, Worry. Risk perception, and Controllability Predict intentions toward COVID-19 preventive behaviors. Front Psychol. 2020;11. 10.3389/fpsyg.2020.582720 PMC7710521 33329239

[18] Bohler-Muller N, Roberts B, Gordon SL, Davids YD (2021). The ‘sacrifice’ of human rights during an unprecedented pandemic: reflections on survey-based evidence. South Afr J Hum Rights.

[19] Kamin T, Perger N, Debevec L, Tivadar B (2021). Alone in a time of pandemic: Solo-Living women coping with physical isolation. Qual Health Res.

[20] Roblain A, Gale J, Abboud S, Arnal C, Bornand T, Hanioti M, et al. Social control and solidarity during the COVID-19 pandemic: the direct and indirect effects of causal attribution of insufficient compliance through perceived anomie. Journal of Community & Applied Social Psychology; 2022. 10.1002/casp.2600 PMC9015481 35463457

[21] Lee HJ, Park BM. Feelings of Entrapment during the COVID-19 pandemic based on ACE Star Model: a Concept Analysis. Healthcare. 2021;9(10). 10.3390/healthcare9101305 PMC8544561 34682983

