# Supplementary material for: Pandemic lockdowns: who feels coerced and why? - a study on perceived coercion, perceived pressures and procedural justice during the UK COVID-19 lockdowns
Source: BMC Public Health. 2024 Mar 13;24:793. doi: 10.1186/s12889-024-17985-1 (PMC10938678; doi:10.1186/s12889-024-17985-1)
Supplement: Supplementary file 1 — Supplementary Material 1 [file 12889_2024_17985_MOESM1_ESM.docx]

**SUPPLEMENTARY TABLES**

**Table S1**: *Participants’ initials according to perceived coercion group*

|  | **Low Perceived Coercion** | **High Perceived Coercion** |
| --- | --- | --- |
| *Initials* | RG | CP |
|  | DB | PS |
|  | SG | ME |
|  | SM | RR |
|  | AA | BB |
|  | RW | MS |
|  | MG | TP |
|  | II | PP |
|  | NN | FM |
|  | DL | RR |
|  | MM |  |
|  | TC |  |
|  | PP |  |
|  | VL |  |
|  | DZ |  |
|  | BE |  |
|  | DD |  |
|  | DP |  |
|  | DA |  |
|  | JB |  |
|  | MA |  |
|  | PE |  |
|  | LK |  |
|  | JA |  |
|  | TH |  |
|  | DG |  |
|  | KA |  |
|  | PS |  |
|  | AH |  |
|  | JJ |  |
